# Anticancer potential of Diospyrin and its analogues: An updated review

**DOI:** 10.1002/fsn3.4237

**Published:** 2024-06-17

**Authors:** Abdur Rauf, Zuneera Akram, Nabia Hafeez, Anees Ahmed Khalil, Ahood Khalid, Hassan A. Hemeg, Abdullah S. M. Aljohani, Waleed Al Abdulmonem, Mohammed Mansour Quradha, Abdulkader Moqbel Farhan Qahtan

**Affiliations:** ^1^ Department of Chemistry University of Swabi Anbar Khyber Pakhtunkhwa Pakistan; ^2^ Department of Pharmacology, Faculty of Pharmaceutical Sciences Baqai Medical University Karachi Pakistan; ^3^ Center of Biotechnology and Microbiology University of Peshawar Peshawar Khyber Pakhtunkhwa Pakistan; ^4^ University Institute of Diet and Nutritional Sciences, Faculty of Allied Health Sciences The University of Lahore Lahore Pakistan; ^5^ Department of Clinical Laboratory Sciences, College of Applied Medical Sciences Taibah University Al‐Medinah Al‐Monawara Saudi Arabia; ^6^ Department of Medical Biosciences, College of Veterinary Medicine Qassim University Buraydah Saudi Arabia; ^7^ Department of Pathology, College of Medicine Qassim University Buraydah Saudi Arabia; ^8^ College of Education Seiyun University Seiyun Hadhramawt Yemen

**Keywords:** anticancer, bioinformatics, chemotherapy, Diospyrin, in vitro, pro‐apoptotic

## Abstract

Cancer, characterized as one of the leading causes of death owing to its heterogeneity and complexity, hence poses a significant challenge to health care system across the globe. Current therapies for cancer curtailment are considered to have associated side effects, therefore discovery of novel alternative approaches is need of the time. In this context, natural products have attained an essential spot in the scientific community for the development of novel cancer treatments. Among others, Diospyrin, a bis‐hydroxy‐naphthoquinonoid, is a vital bioactive component present in various *Diospyros* and *Euclea* species. The bioactivity associated with Diospyrin's makes it a promising “lead molecule” for new chemotherapy. In this review, biosynthesis of Diospyrin and its analogues along with their anticancer activities has been discussed. Moreover, this review briefly discusses probable modes of action of Diospyrin and its analogues by targeting the molecular signal transduction pathways. This review also highlights the toxicological and clinical implications of diospyrin and its derivatives. Further pharmacological and pharmacogenetic studies are required to better understand the anticancer potential of Diospyrin and its analogues at the molecular and genetic levels.

## Graphical abstract

Numerous analogues and derivatives have been synthesized, and their pro‐apoptotic and anti‐cancer activities have been investigated. This review focuses primarily on the bioactivity of Diospyrin and its analogues/derivatives and the likely processes involved.

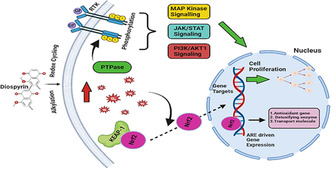

## 1 INTRODUCTION

Historically, using natural products has considerably aided the development of new cancer treatments. Multiple analyses and evaluations have emphasized the importance of natural ingredients in pharmaceutical research (Atanasov et al., 2021; Jones et al., 2006; Koehn & Carter, 2005; Newman et al., 2000, 2003; Paterson & Anderson, 2005), and approximately 200,000 natural compounds have been identified (Nakanishi, 2005). In the 1940s, 47% (73/155) of small molecules identified in anticancer therapies originated directly or indirectly from natural sources (Newman & Cragg, 2007). Due to their chemical diversity and high chiral center content, natural compounds are a starting point for developing new drugs.

Bioactivity‐screening methods and the isolation of bioactive compounds are presently the cornerstones of drug development from plants (Potterat & Hamburger, 2006). Compounds derived from plants, microorganisms, animals, and marine are frequently used as “lead” molecules that can be optimized for increased activity, decreased toxicity, and improved pharmacokinetics. Several plant‐derived compounds with a quinone pharmacophore have been identified; these compounds are prospective “leads” for the chemotherapeutic treatment of various cancers. These substances include paclitaxel, camptothecin, podophyllotoxin, vinblastine, vincristine, and docetaxel. The Quinone compounds are the second most widely utilized group of cancer medications (Sanchez‐Cruz & Alegría, 2009). Secondary metabolites are ubiquitous and frequently play a vital role in the biochemistry of energy production and the electron transport chain. It has been demonstrated that certain plant‐based naphthoquinonoids inhibit the proliferation of cancer cell lines. Among these are doxorubicin, β‐lapachone, plumbagin, and shikonin. These chemicals cause cancer cells to undergo apoptosis. Two hypothesized mechanisms for such drugs' cytostatic and antiproliferative effects are redox cycling and reductive alkylation (Sagar et al., 2010). Several naturally occurring quinolones tend to produce intracellular reactive oxygen species (ROS), which contributes to their anticancer effects by inducing apoptosis.

Diospyrin diethyl ether (D7) is a bisnaphthoquinonoid derivative isolated from numerous *Diospyros* species' heartwood. It has been shown to induce apoptosis in response to oxidative stress in human cancer cells and animal models of the disease, suggesting that it may have anticancer properties (Kumar et al., 2012). Kapil and Dhar isolated and characterized the chemical properties of diospyrin from *Diospyros montana* Roxb. (Ebenaceae) in 1961 (Hussain et al., 2015; Sagar et al., 2010). Ganguly and Govindachari postulated in 1966 that the structure of diospyrin consisted of a dimer of 7‐methyljuglone coupled to C‐2 and C‐3. Later, structure of diospyrin was modified by adding a C‐2 to C‐6 linkage (Hussain et al., 2015). Harrison and Musgrave (2004) conducted crystallographic research that revealed diospyrin to be 2,6'‐bis (5‐hydroxy‐7‐methyl‐1,4‐naphthoquinone). According to the hypothesized structure, diospyrin is optically inactive, so there is no rotational restriction around the connecting bonds (Yoshida & Mori, 2000). *Diospyros*, a genus of Ebenaceae plants, has been reported as the source of diospyrin isolation (Hussain et al., 2015). Since it was initially discovered that diospyrin inhibited the growth of tumors, it has been used as a “lead molecule” in the research and development of treatments for cancer. Because of this, the synthesis of diospyrin derivatives and analogues has attracted a lot of attention, and a good number of these compounds have been tested to see whether they exhibit features conducive to apoptosis (Harrison & Musgrave, 2004).

The main aim of this review is to provide an insight into biosynthesis of Diospyrin and its analogues along with their anticancer activities. We have also highlighted the anticancer mechanisms of Diospyrin and its analogues to show how they interfere with crucial signal transduction pathways within cancer cells. These pathways are intricate networks of molecular signals that regulate cellular processes, such as growth, proliferation, differentiation, and apoptosis (programmed cell death). In the end, we have also briefly discussed the toxicological and clinical implications associated with diospyrin and its derivatives.

## 2 BIOSYNTHESIS OF DIOSPYRIN AND ITS ANALOGUES

**FIGURE 1:**
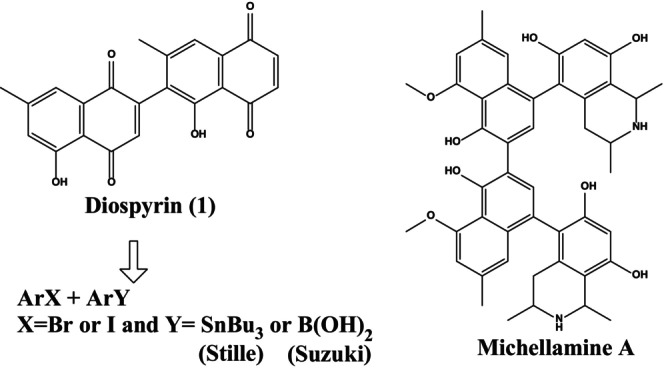
Diospyrin's structure and retrosynthetic evaluation.

In 2000, Yoshida and Mori proposed the retrosynthetic degradation of diospyrin shown in Figure 1 (Yoshida & Mori, 2000). The “Stille reaction via organotin intermediates” (Farina et al., 2004) and the “Suzuki coupling via organoboronic acid intermediates” (Miyaura & Suzuki, 1995) are viable methods for synthesizing the two naphthoquinone moieties and joining them. Recent efforts to synthesize michellamine A and related compounds (Bringmann et al., 1998; de Koning et al., 1999; Hobbs et al., 1997; Hoye et al., 1999) taught us about the synthesis.

**FIGURE 2:**
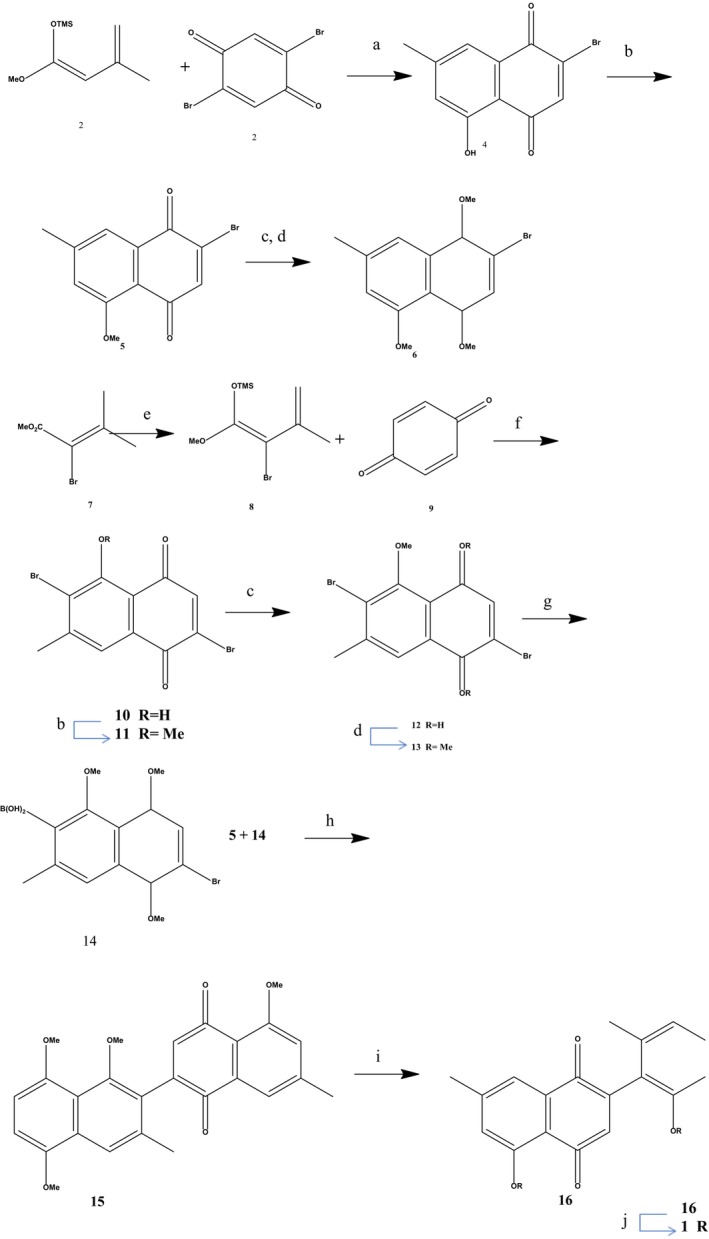
Synthesis of Diospyrin.

Figure 2 is a condensed version of Yoshida and Mori's original diospyrin (1) synthesis (Yoshida & Mori, 2000). The naphthalene precursors 6 and 13 were made in the first step of the synthesis. To obtain 4, the widely recognized 1‐methoxy‐3‐methyl‐1‐trimethylsilyloxy‐1,3‐butadiene (2) was reacted with 2,5‐dibromo‐1,4‐ benzoquinone (3) through Diels–Alder reaction (Casey et al., 1981). To synthesize 5, methyl iodide and silver oxide were methylated, and then the resulting naphthalenediol was reduced with tin (II) chloride and subjected to yet more methylation to produce 6 (Kesteleyn et al., 1999). To yield 13, another Diels–Alder reaction was carried out. To achieve this, 2‐bromo‐1‐methoxy‐3‐methyl‐1‐trimethylsilyloxy‐1,3‐butadiene (8) was synthesized using the known methyl 2‐bromosenecioate (7) (Martin & Caputo, 1974). Following a Diels–Alder reaction in dichloromethane between 8 and 1,4‐benzoquinone (9), the resultant mixture was treated with weak hydrochloric acid (HCl) to eliminate the partially residual trimethylsilyl group, yielding a combination of products 10 and 11. To afford pure 11, this was methylated completely with methyl iodide and silver oxide. Reducing 11 with tin (II) chloride yielded 12, which was subsequently methylated to yield 13. Yoshida and Mori attempted to obtain the corresponding tributyltin derivatives by lithiation and then stannylating 6 or 13. The attempt to combine the two naphthalene units was abandoned, as the Stille reaction proved ineffective (Yoshida & Mori, 2000). Suzuki coupling, whose application in phytoalexin synthesis was recently reported by Takikawa et al. (1999), was the apparent alternative strategy (Miyaura et al., 1981; Yoshida & Mori, 2000). By lithiating 13 and then reacting it with trimethyl borate, the desired boronic acid 14 was produced. A 66% yield of 14 could be obtained by removing the accompanying impurities with heated hexane. The coupling of 14 with 6 in the presence of tetrakis(triphenylphosphane)palladium (0) failed when four different bases (sodium carbonate, cesium carbonate, barium hydroxide, and potassium phosphate) were used. We were able to couple bromo‐1,4‐ naphthoquinone (5) and (14) with a 53% yield using tetrakis(triphenylphosphane)palladium (0) as the catalyst in a solution of sodium carbonate, yielding orange‐red needles. Diospyrin dimethyl ether (16), a yellow needle with a melting point of 256–258°C, was obtained through the oxidative demethylation of 15 with ceric ammonium nitrate (Kesteleyn et al., 1999; Syper et al., 1979). Only aluminum chloride in dichloromethane at room temperature could demethylate 16 (Sarma et al., 2008). Based on 2 (5 stages) or 7 (9 steps), the aggregate yield of diospyrin (1) was 19% and 9%, respectively (Yoshida & Mori, 2000).

Diospyrin was first converted to its dimethyl ether derivative using methyl iodide and silver oxide to develop analogues with aliphatic and aromatic amino substituents. This allowed for the generation of analogues. These compounds have distinct reaction conditions optimal for the standard 1,4‐Michael addition and subsequent air oxidation (Sidhu & Prasad, 1967). The glycoside derivative mannosyl adduct 18 was identified as a novel “lead” chemical by Das Sarma et al. This compound is derived from diospyrin (Sagar et al., 2010). Sidhu and Prasad (1967) first isolated diospyrin analogues such as isodiospyrin from *Diospyros chloroxylon* Roxb (Fallas & Thomson, 1968). This asymmetrical dimer of 7‐methyljuglone was also discovered in other *Diospyros* species (Lajubutu et al., 1995). Diosquinone is assumed to be the first quinone epoxide isolated from a root of *Diospyros mespiliformis* (O'brien, 1991).

## 3 ANTICANCER POTENTIAL

Natural compounds have played a crucial role in searching for and developing anticancer drugs. Cancer‐fighting quinonoid pharmaceuticals can produce ROS, presumably via a redox cycling mechanism. In vivo bioreduction of these compounds initiates the production of ROS, which interacts with the nucleic acids of tumor cells, resulting in cytotoxicity (Johnson et al., 1999). Recent attempts have been made to correlate quinonoid activity with their redox potential (Chakrabarty et al., 2002). Diospyrin inhibited Ehrlich ascites carcinoma (EAC) in rodents, and its derivatives demonstrated cytotoxicity (Sarma et al., 2007). Table 1 shows studies and their results revealing anticancer properties of Diospyrin.

**TABLE 1:** Anticancer potential of Diospyrin and their derivatives.

Phytochemical	Type of cancer	Cell line	Outcomes	References
Diospyrin ethyl ether	Cervical cancer	HeLa	50 μM	Hazra et al. (2007)
	Chronic myelogenous leukemia	K‐562	40 μM	
	Promyelocytic leukemia	HL‐60	30 μM	
	Breast cancer	MCF‐7	50 μM	
Quinonoid analogues of diospyrin	Osteosarcoma	MG‐63	35% (APOpercentage)	Sagar et al. (2010)
	Cervical cancer	HeLa	3% (APOpercentage)	
	Breast cancer	MCF‐7	65% (APOpercentage)	
Isodiospyrin	Colon malignancy	HCT‐8	14.77 μg/mL	Kuo et al. (1997) and Ting et al. (2003)
	Colon carcinoma	COLO‐205	2.24 μg/mL	
	Lymphocytic leukemia	P‐388	0.851 ± 0.050 μg/mL	
	Nasopharyngeal carcinoma	KB	1.81 μg/mL	
	Hepatoma	HEPA‐3B	1.72 μg/mL	
	Cervical cancer	HeLa	5.55 μg/mL	
Glycoside derivative of diospyrin	Epidermoid laryngeal carcinoma	Hep 2	0.26 ± 0.05 μM	De Araújo et al. (2014)
	Malignant skin melanoma	A375	0.02 ± 0.01 μM	
Epoxide derivative	Epidermoid laryngeal carcinoma	Hep 2	0.2 ± 0.05 μM	Sagar et al. (2010)
	Malignant skin melanoma	A375	0.03 ± 0.01 μM	

**FIGURE 3:**
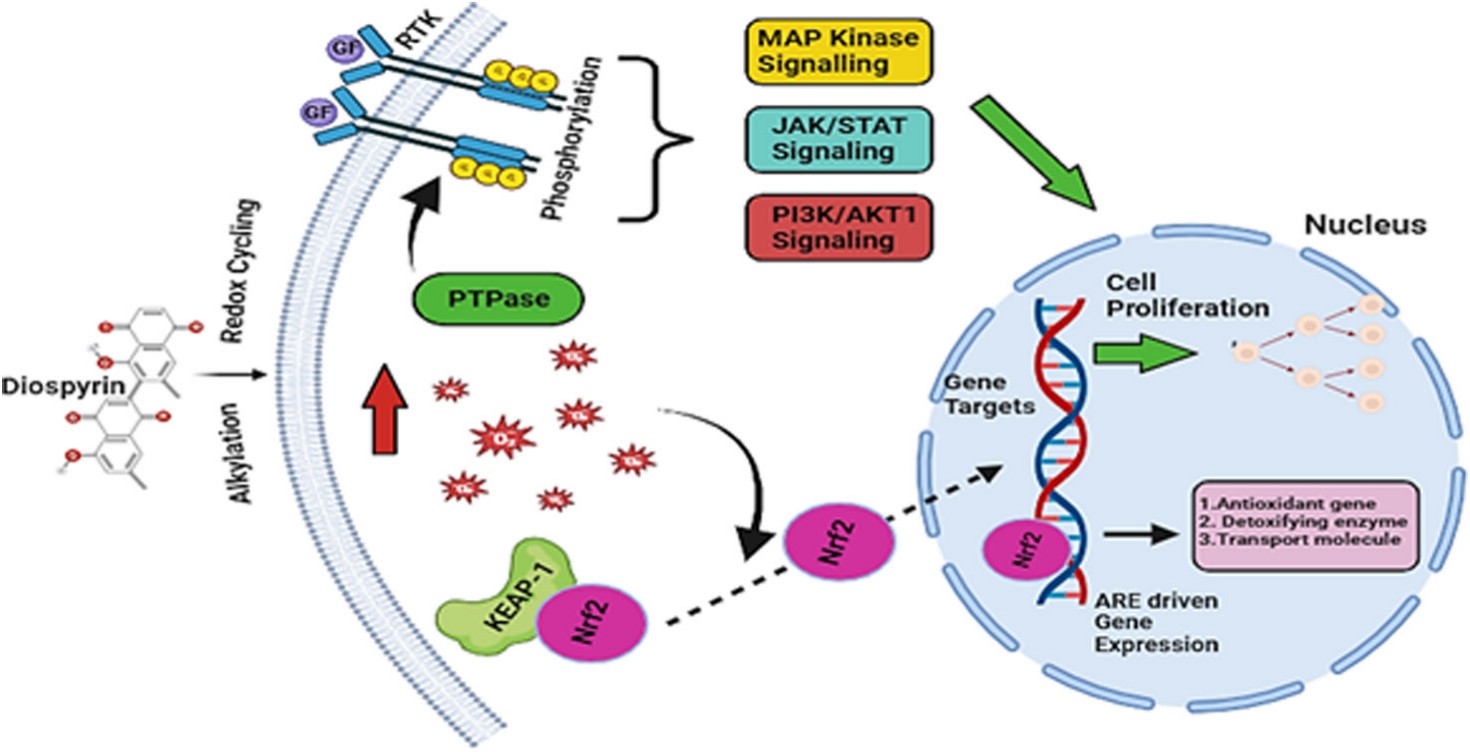
Proposed anticancer properties of Diospyrin.

Numerous laboratories have devoted the last quarter century to researching diospyrin and its derivatives due to its potential as anticancer lead compounds. Several derivatives and analogues with pro‐apoptotic and anticancer properties have been developed and tested. Sagar et al. provided an updated summary of the research on the anticancer effects of diospyrin and its derivatives/analogues. Recently, Diospyrin and its analogue (8‐hydroxydiospyrin) have been examined for their potential antitumor and anti‐inflammatory properties. Both diospyrin (IC50: 47.40 ppm) and 8‐hydroxydiospyrin (IC50: 36.91 ppm) have reported significant cytotoxic activity, as investigated by Rauf et al. (2021). Both these compounds showed IC50 values of 426 and 412 ppm (parts per million), respectively, as examined by Epstein–Barr‐Virus (EVA) early antigen activation assay. At a concentration of 1000 (molar ratio 32 pmol of 12‐O‐tetradecanoylphorbol‐13‐acetate [TPA]), diospyrin and 8‐hydroxydiospyrin revealed a 60% survival rate of lymphoblastoid Raji cells. Moreover, in vivo experimentation demonstrated that diospyrin and 8‐hydroxydiospyrin helped in delaying the development of papillomas on mouse skin. Purposely, both the compounds demonstrated 50% effect post 13th and 14th weeks of administration, respectively. Whereas, maximum (100%) effect was noticed at the 20th week of administration (Rauf et al., 2021). Diospyrin diethyl ether, a derivative of diospyrin, increased the cytosolic calcium [Ca^2+^]c causing apoptotic cellular death that was triggered due to the administration of the compound in human breast cancer cells (MCF‐7). The anticancer effects including the induction of apoptotic conditions were momentously retarded due to pretreatment of a cell‐permeable Ca^2+^−specific chelator (Bapta‐AM) or a calpain inhibitor (calpeptin). Moreover, Diospyrin diethyl ether‐induced cytosolic calcium [Ca^2+^]c was reported in alteration of mitochondrial membrane potential and induced the release of cytochrome c that was retarded by Bapta‐AM. Conclusively, outcome of this experimentation provides an insight regarding the accentuating effect of Diospyrin diethyl ether‐induced Ca^2+^ on apoptotic mitochondrial pathways (Kumar et al., 2012). Further analysis of amino naphthoquinone derivatives of the diospyrin, isolated from *Diospyros montana* Roxb., was performed to elucidate their ability to prevent tumor growth. An amino acetate derivative significantly increased life span in vivo and had the lowest IC50 in vitro when used to treat mice EAC. Figure 3 shows proposed anticancer properties of Diospyrin. The exact analogue showed noticeably improved antiproliferative activity when tested against human cell lines, including malignant skin melanoma and epidermoid laryngeal carcinoma, compared to the natural precursor, diospyrin. In addition, it was demonstrated that the cytotoxicity of diospyrin and all of its derivatives against tumor cells was approximately 17–1441 times greater than that of normal human lymphocytes. These quinonoids generated significant levels of ROS in EAC cells, roughly corresponding to their IC50 values (De Araújo et al., 2014).

### 3.1 Diospyrin and its analogues'‐signal transduction pathways within cancer cells

As mentioned in the previous heading, Diospyrin and its analogues have indeed garnered significant attention in the realm of cancer research due to their potential anticancer properties. These compounds, derived from natural sources like the *Diospyros* genus of plants, have shown promise in inhibiting cancer cell growth through various molecular mechanisms.

One of the primary mechanisms through which Diospyrin and its analogues exert their anticancer effects is by interfering with crucial signal transduction pathways within cancer cells. These pathways are intricate networks of molecular signals that regulate cellular processes, such as growth, proliferation, differentiation, and apoptosis (programmed cell death).

Here's a general overview of some of the key signal transduction pathways targeted by Diospyrin and its analogues:

#### 3.1.1 NF‐κB pathway

Understanding the nuclear factor kappa‐light‐chain‐enhancer of activated B cells (NF‐κB) pathway is crucial for comprehending how it regulates immune responses, inflammation, and cell survival (Matsumori, 2023). Disruption of NF‐κB signaling is commonly seen in different types of cancers, leading to the promotion of tumor growth and the development of resistance to apoptosis. Research has demonstrated that Diospyrin and similar compounds can effectively hinder NF‐κB activation, leading to apoptosis and the inhibition of tumor growth (Rauf et al., 2021).

#### 3.1.2 MAPK/ERK pathway

The mitogen‐activated protein kinase/extracellular signal‐regulated kinase (MAPK/ERK) pathway plays a crucial role in transmitting extracellular signals to the nucleus, where they regulate gene expression involved in cell proliferation and survival. Dysregulation of this pathway is common in cancer and is associated with uncontrolled cell growth (Park, 2023). Diospyrin and its analogues have been reported to modulate the MAPK/ERK pathway, leading to the inhibition of cancer cell proliferation and induction of apoptosis (Kim et al., 2020).

#### 3.1.3 PI3K/Akt/mTOR pathway

The phosphatidylinositol 3‐kinase (PI3K)/protein kinase B (Akt)/mammalian target of the rapamycin (mTOR) pathway is a key signaling cascade involved in cell growth, proliferation, and survival (Basnet et al., 2024). Aberrant activation of this pathway is implicated in various cancers, promoting tumor progression and resistance to therapy (El‐Tanani et al., 2023). Diospyrin and its analogues have been shown to inhibit PI3K/Akt/mTOR signaling, leading to suppression of cancer cell proliferation and induction of apoptosis. Diospyrin reduces nitric oxide (NO) production and intracellular calcium release, and inhibits p38 MAPK and extracellular signal‐regulated protein kinase 1 and 2 (ERK1/2) phosphorylation (Kim et al., 2020; Shahidullah et al., 2020).

#### 3.1.4 Wnt/β‐catenin pathway

The Wnt/β‐catenin pathway plays a crucial role in regulating cell proliferation, differentiation, and stem cell maintenance. Dysregulation of this pathway is associated with the development and progression of various cancers (He & Gan, 2023). Diospyrin and its analogues may interfere with Wnt/β‐catenin signaling, thereby inhibiting cancer cell growth and metastasis (Sarma et al., 2007).

## 4 TOXICOLOGICAL PROFILE

By reacting lawsone with various aromatic and aliphatic aldehydes in an atmosphere that was only weakly acidic, a total of eight substituted bis‐2‐hydroxy‐1,4‐naphthoquinone derivatives were generated. Six compounds exhibited acceptable antileishmanial activity without causing significant adverse effects when their antileishmanial activity was evaluated (Xiu‐Zhen et al., 1989). Epoxide derivative showed much lower toxicity to peripheral blood mononuclear cells (PBMCs). Hence, it was proposed as a new ‘lead’ molecule for cancer treatment.

## 5 CLINICAL IMPLICATIONS

On four distinct human tumor cell lines (HeLa [Cervical Cancer], K‐562 [chronic myelogenous leukemia], HL‐60 [promyelocytic leukemia], and MCF‐7 [breast cancer]), the cytotoxic properties of diospyrin compounds were evaluated. In screening experiments, Diospyrin diethyl ether outperformed diospyrin and its other derivatives. For potential application in future clinical trials, the effect of Diospyrin and Diospyrin diethyl ether was studied in greater depth (Hazra et al., 1984, 2007). Sagar et al. (2010) researched three unique cancer cell lines: MG‐63, HeLa, and MCF‐7 (human osteosarcoma), using four distinct quinonoid analogues of diospyrin. Isodiospyrin has cytotoxic effects on cancer cell lines. According to previous studies, isodiospyrin has exhibited significant cytotoxicity against the HCT‐8 colon malignancy, COLO‐205 colon carcinoma, P‐388 lymphocytic leukemia, KB (nasopharyngeal carcinoma), HEPA‐3B hepatoma, and HeLa cervical carcinoma (Kuo et al., 1997; Ting et al., 2003). Diospyrin, a member of a new class of DNA topoisomerase poisons, is an isoform‐stable inhibitor of *Leishmania donovani* type I DNA topoisomerase I. Activity results for Isodiospyrin were comparable to those for Diospyrin. Using the MTT (3‐(4,5‐dimethylthiazol‐2‐yl)‐2,5‐diphenyltetrazolium bromide) cytotoxicity assay, it was discovered that a glycoside derivative of diospyrin was the most active of all the glycoconjugate derivatives that were tested against two human cancer cell lines: epidermoid laryngeal carcinoma (Hep2) and malignant skin melanoma (A375), as well as nonmalignant peripheral blood mononuclear cells (PBMCs) (De Araújo et al., 2014). Against the Hep2 and A375 cell lines, the epoxide derivative showed the most promising results (Sagar et al., 2010).

## 6 CONCLUSIONS AND FUTURE PERSPECTIVES

Although diospyrin and its derivatives have demonstrated apoptotic activity in the laboratory, insufficient data have been gathered to understand the mechanisms underlying their anticancer activity. Initial investigations point to the possibility that reactive oxygen species (also known as ROS) are an essential factor in the antitumor activity of diospyrin and its derivatives. It has been shown that ROS production's origin and location can affect cell survival and proliferation. Lysosomes and autophagosomes are two additional sites where ROS are produced. Investigating how diospyrin and its derivatives affect ROS production in diverse cellular compartments could prove to be a fascinating challenge. Other pharmacological and pharmacogenetic studies are required to understand this lead compound's bioactivity mechanisms at the cellular and genetic levels.

## AUTHOR CONTRIBUTIONS

**Abdur Rauf:** Conceptualization (equal); data curation (equal); investigation (equal); methodology (equal); supervision (equal); validation (equal); writing – review and editing (equal). **Zuneera Akram:** Data curation (equal); formal analysis (equal); methodology (equal); resources (equal); validation (equal); writing – review and editing (equal). **Nabia Hafeez:** Formal analysis (equal); investigation (equal); methodology (equal); validation (equal); writing – original draft (equal). **Anees Ahmed Khalil:** Conceptualization (equal); formal analysis (equal); methodology (equal); project administration (equal); supervision (equal); writing – original draft (equal); writing – review and editing (equal). **Ahood Khalid:** Data curation (equal); formal analysis (equal); validation (equal); visualization (equal); writing – original draft (equal); writing – review and editing (equal). **Hassan A. Hemeg:** Conceptualization (equal); formal analysis (equal); methodology (equal); validation (equal); writing – original draft (equal). **Abdullah S. M. Aljohani:** Investigation (equal); methodology (equal); visualization (equal); writing – review and editing (equal). **Waleed Al Abdulmonem:** Data curation (equal); investigation (equal); methodology (equal); validation (equal); writing – original draft (equal); writing – review and editing (equal). **Mohammed Mansour Quradha:** Data curation (equal); investigation (equal); methodology (equal); resources (equal); supervision (equal); visualization (equal); writing – original draft (equal). **Abdulkader Moqbel Farhan Qahtan:** Data curation (equal); investigation (equal); methodology (equal); validation (equal).

## CONFLICT OF INTEREST STATEMENT

The authors declare no conflicts of interest.

## Data Availability

The dataset supporting the conclusions of this article is included within the report.
